# Biofilm and Dental Biomaterials

**DOI:** 10.3390/ma8062887

**Published:** 2015-05-25

**Authors:** Marit Øilo, Vidar Bakken

**Affiliations:** 1 Department of Clinical Dentistry, Faculty of Medicine and Dentistry, University of Bergen, Aarstadveien 19, Bergen NO-5009, Norway; 2 Department of Clinical Science, Faculty of Medicine and Dentistry, University of Bergen, Jonas Lies vei 65, The Laboratory Building, Bergen NO-5021, Norway; E-Mail: Vidar.Bakken@k2.uib.no

**Keywords:** biofilm, biomaterials, dental materials, metals, polymers, ceramics

## Abstract

All treatment involving the use of biomaterials in the body can affect the host in positive or negative ways. The microbiological environment in the oral cavity is affected by the composition and shape of the biomaterials used for oral restorations. This may impair the patients’ oral health and sometimes their general health as well. Many factors determine the composition of the microbiota and the formation of biofilm in relation to biomaterials such as, surface roughness, surface energy and chemical composition, This paper aims to give an overview of the scientific literature regarding the association between the chemical, mechanical and physical properties of dental biomaterials and oral biofilm formation, with emphasis on current research and future perspectives.

## 1. Introduction

Dental treatment involving biomaterials will alter the mechanical, physiological and chemical conditions in the oral cavity. The degree of change depends on the size and quality of the restoration inserted, which in turn, will affect the microbiology of the oral cavity. All surfaces in the oral cavity are covered by a pellicle of glycoproteins from saliva within seconds after cleaning [1,2]. Microorganisms are able to adhere to this layer and form biofilms organized as a consortium of bacteria, virus and fungi. Most of the microorganisms present in the oral biofilm are harmless natural inhabitants, but some have the potential to cause damage to the mineralized tissues or infections in the soft tissues. Primary infections (caries) are responsible for less than half of the dental restorations produced annually [3]. Most of the restorations placed in general dental practices are replacements of old restorations, and the majority of these need replacement because of biofilm related secondary infections. By inserting foreign bodies such as dental restorations, new niches appear for the microorganisms. Such niches create areas promoting biofilm accumulation with a pathological potential. Increased numbers of microorganisms with a pathological ability will increase the risk of developing disease. Examples of such diseases are secondary caries along a restoration, fungal infections in relation to complete dentures or progression of periodontal disease on teeth supporting a partial denture.

However, patients with active caries, worn or broken fillings or badly fitting dentures, may reduce the pathological potential of the biofilm with new, optimally shaped dental restorations. The aim of using biomaterials for treating patients is to do more good than harm. The biomaterials’ ability to inhibit biofilm formation is an important factor for clinical success [4]. There is a global interest in finding ways to reduce the biofilm formation on biomaterials in general. Biofilm infections of medical devices at other body sites than the oral cavity also constitute major problems [5].

## 2. Oral Biofilm

In the human oral cavity there are hundreds of different species of microorganisms, including bacteria, virus and fungi [6]. More than 700 unique bacterial species have been detected [7]. There can be more than 10^11^ microorganisms per mg of dental plaque [7,8]. These live in complex societies, usually organized in thin layers covering the oral surfaces—biofilm—for example as dental plaque on tooth surfaces [1,2]. Immediately after cleaning, the proteins from saliva will cover the tooth surfaces in a pellicle. Bacteria attach to this pellicle by microfilaments in their cell walls. When the bacteria increase in number, they will be able to communicate by secreting signal molecules and create a community [1,9]. The bacteria secrete proteins, polysaccharides, nucleic acids and other substances to the extracellular matrix, additionally containing proteins and nutrients from saliva. This matrix is the “glue” of the biofilm.

The microorganisms inside a matrix behave differently from bacteria floating freely in the saliva (planktonic growth). The biofilm community behaves as a unit in response to environmental changes rather than as single bacterial responses. The matrix protects the organisms inside from chemical treatment that could have been lethal for planktonic bacteria, for example antibacterial mouth rinses [10]. Complete removal of oral plaque is difficult due to limited access between teeth and in deep crevices on tooth surfaces. Dental restorations with pores, gaps and margins further complicate this. In healthy individuals the biofilm on teeth will function as a protective shield from foreign microorganisms and chemicals in the food, such as acids that can potentially dissolve dental enamel [11]. If, however, the biofilm is left untreated, an ecological shift might occur, favoring microorganisms that may have detrimental effects on teeth, surrounding tissues and the patient’s oral and general health.

The thickness and structure of a biofilm will be affected by many factors, such as pH, nutrients, oxygen, time since last cleaning and the kind of surface to which it is attached [2,12,13,14,15,16,17]. The biofilm in the oral cavity will therefore differ in the different locations, such as on the cheek or in between teeth. A biofilm that is allowed to grow over days will have a different composition than a biofilm that is mechanically removed and renewed daily [2]. The bacteria adjust to the matrix and surrounding organisms by gene regulations. Biofilm formation is a big problem for all medical biomaterials in humid environments, such as artificial heart valves, artificial vocal cords and incubation tubes [5,18]. They often have the added complications that access for cleaning procedures is impossible. However, they are usually not in environments with such an abundance of microorganisms as in the oral cavity.

## 3. Oral Microorganisms and Oral Infections

Growth of pathogenic microorganisms can lead to disease if the host’s immune response is not able to neutralize or destroy them [9]. The most common oral infections are pulpitis from untreated caries, gingivitis, and periodontitis [2]. The microorganisms in oral biofilm are mainly bacteria. However, adults usually have both fungal and viral species present in the oral microflora [6,7]. *Candida albicans* will normally be present only in small amounts in healthy adults because the healthy biofilm favor other microorganisms. The microflora develops gradually as the environment in the mouth changes. Microorganisms with and without known ability to provoke diseases will be present in the flora. As long as the majority of microorganisms are non-pathogenic, the host stays healthy, but if the conditions favor growth of pathogens, disease might develop [7].

**Figure 1:**
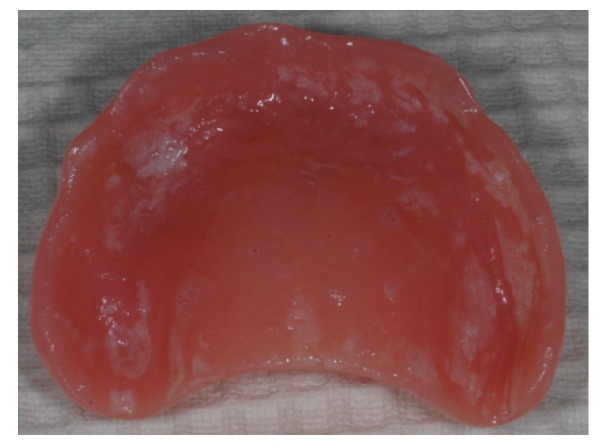
Biofilm on a denture belonging to a patient with poor oral hygiene routine. (photo: Marit Øilo).

Teeth are unique organs since they penetrate the oral mucosa and have one part secured in bone and one part exposed to the oral cavity [19]. The exposed part is continually colonized by the oral microorganisms. The periodontal pocket surrounding the tooth is designed as a protective barrier against the invasion of microorganisms to the mucosa and the alveolar bone. The cells in the pocket release exudate with antibacterial effect that hinders microorganisms from invading the pockets. Increased thickness of the biofilm will reduce the pH-, oxygen- and nutrient-levels in the bottom layers of the plaque (Figure 1) [2,9,12,17,20]. Food debris in the biofilm will ensure access to nutrients for the bacteria. This will be favorable for the species associated with dental caries (*Streptococcus mutans*, *Actinomyces spp.*, *Veillonella* *spp.*, *Lactobacillus* *spp.*) and gingival and periodontal inflammation (*Porphyromonas gingivalis*, *Treponema denticola*, *Tannerella forsythia etc*.)*.*

Caries is in itself not an infection, but demineralization of dentin and enamel. The demineralization occurs as a consequence of acid production from bacteria in the biofilm when they process sugars in plaque [21]. The biofilm becomes acidic (pH < 5) and the hydroxyapatite in the enamel dissolves. When the lesion is very deep, the pulp of the tooth might be infected with oral bacteria and inflammation occurs, known as pulpitis [2]. Gingivitis occurs when the biofilm is too thick to be effectively washed away by the gingival exudate. The anaerobic bacteria are favored and intrude into the periodontal pocket. Proliferation of pathogenic bacteria in the pocket induces inflammatory response in the gingiva with swelling and increased bleeding which complicates the cleaning procedures. This response can in turn dissolve the alveolar bone supporting the teeth—periodontitis—which eventually will result in tooth loss [1,2,6,12].

**Figure 2:**
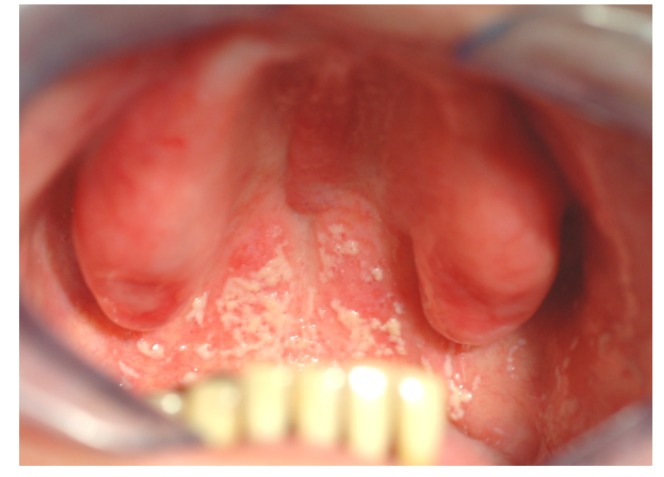
Patient with denture stomatitis. The infection has spread from the denture covered palate to the throat and stomach. The patient experienced no oral pain and was unaware of the situation (photo: Marit Øilo).

Oral infections can affect the hosts’ general health in many ways [8,22]. Firstly, it is unfavorable to have ongoing infections in general. Oral infections such as gingivitis can involve quite large areas, and is a constant burden for the hosts’ immune system. This is, of course, most harmful for patients with other ongoing inflammations or diseases. Secondly, oral microbes may spread to other organs within the host either via the respiratory system or through the blood stream [1,2,8,20,22,23]. This is, again, most harmful for patients with other complications, such as artificial heart valves, or transplanted organs. Elderly people with pneumonia will often have oral microbes in their lungs, which may have been aspired from the oropharynx and caused the inflammation in the lungs [8,20,23]. The direct causality between oral infections and systemic disease has been difficult to prove. It is difficult to detect whether the oral microorganisms were present prior to the disease and were the direct cause of the infection or not. It is also possible that the oral microbes entered the loci after the primary inflammation occurred due to reduced effect in the immune response system. *Candida albicans* can spread directly from the oral cavity to the throat and stomach (Figure 2) [24,25]. General candida infections are extremely difficult to cure due to the high risk of re-colonization. Thirdly, individuals with poor general health will normally also have poor oral health, due to malfunctioning immune system and altered chemical and physical conditions in the body, such as reduced saliva flow, iron deficiency, malnutrition, medical treatment, *etc*. [26,27]. Sick and elderly have reduced ability to clean their teeth properly and are therefore more exposed to development of unfavorable biofilm and subsequent general infection [28].

## 4. Biomaterials’ Effect on Biofilm

**Figure 3:**
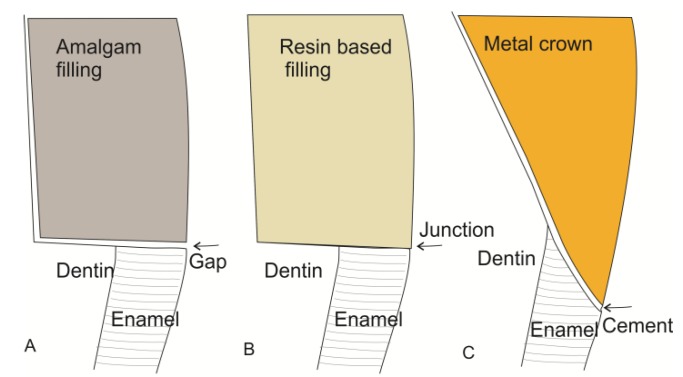
Different biomaterials create different types of junctions between tooth and restoration. The illustrations represent cross sections of the tooth-restoration border. (**A**) An amalgam filling is not directly attached to the tooth. The gap is a potential area for ingrowth of bacteria which causes secondary caries; (**B**) The resin based fillings are supposed to be adhesively attached to the tooth substance with no gaps in between. This requires that the filling is placed and cured with a special technique to avoid gaps caused by polymerization shrinkage; (**C**) A crown is fixed to the tooth by means of cement which fills the gap between tooth and restoration. This will always cause irregularities on the surface. The exposed cement is subjected to wash out and wear causing an increase of the groove over time.

**Figure 4:**
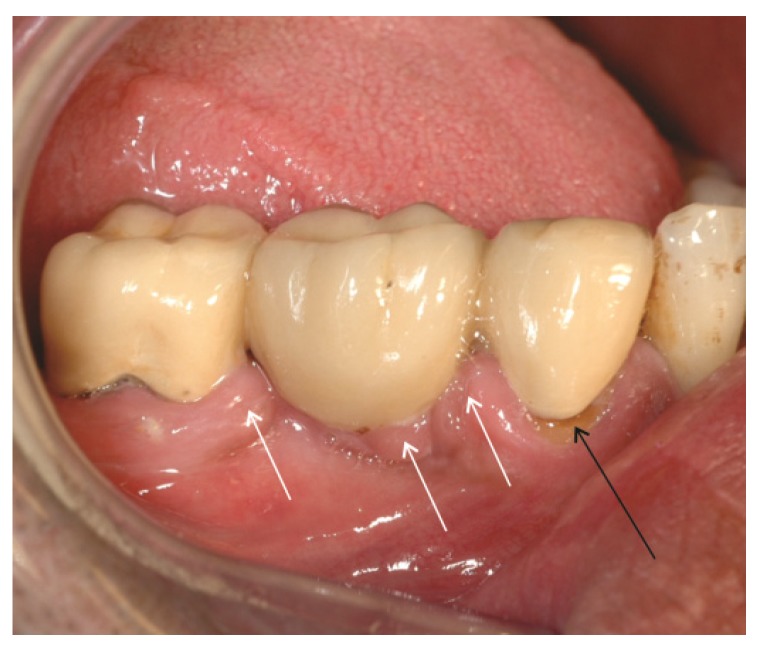
Secondary caries at the crown margin (black arrow) caused by accumulation of biofilm in the crevice between crown and tooth. Hyperplastic gingiva (white arrows) due to increased plaque accumulation in the connector area of the fixed partial denture. The connector area of the bridge has a bulky design which complicates interdental hygiene (photo: Marit Øilo).

Dental restorations affect the composition of the biofilm in many ways. There will always be steps, gaps or groves between tooth and restoration (Figure 3). These will complicate mechanical biofilm removal and alter the chemical balance in the biofilm in the region (Figure 4) [29,30]. Restorations differ from enamel with regard to surface roughness, surface energy and chemical composition [29,30,31,32,33]. Most adults have at least one dental restoration and the role of biofilm related infections related to the restoration as opposed to primary oral infections is not easily distinguished.

Many different studies have been performed evaluating the effect on biofilm formation from surface qualities such as surface energy, roughness, topography and chemical composition of the restorative materials [34,35,36]. Both surface quality and chemical composition will affect the topography and surface energy and the different studies do not always disclose or differentiate properly between the different factors. Multiple factors are working simultaneously that all may affect the outcome. Comparison between different studies and among materials is not straight forward. Furthermore, the findings from *in vitro* and *in vivo* studies are not always in coherence with each other, which indicates that the oral biofilms *in vivo* are complicated and difficult to mimic in a laboratory setting. Most studies evaluate only a selection of pathogenic bacteria or only one surface variable [7,21]. As discussed above, it is not likely that one surface quality or single microorganisms are the cause of disease alone but rather a shift in the composition in the biofilm.

### 4.1. Surface Energy

Surfaces with a low surface energy usually display lower adherence to biofilms than similar surfaces with higher surface energy [35]. No effect of changes in surface energy was found in a study on surface nanoroughness, texture and chemistry [37]. Most dental materials, with the exception of ceramics, have a higher surface energy than enamel and have thus a greater risk of biofilm accumulation. Alteration in surface roughness will in most cases also alter the surface energy. It is therefore difficult to distinguish between the two factors. It seems that surface roughness plays a more important role than surface energy [36].

### 4.2. Surface Roughness

Increased surface roughness and complicated topography shows higher affinity to microbes than smoother surfaces and subsequently increased difficulty in complete removal of the biofilm by mechanical brushing [15,34,35,38]. The surface roughness seems to affect only the number of bacteria in the biofilm, not the species. A rough surface increases the surface area available for colonization compared to a smooth surface [36]. Furthermore, the crevices created by the roughness generate shelters for the bacteria so they have time for securing their attachment to the pellicle. On smooth surfaces, the brushing of tongue or cheek movement is sufficient for mechanical removal of the biofilm. A maximum surface roughness of R_a_ = 0.2 µm has been suggested as a threshold value for bacterial retention. Below this value, no further reductions were observed, while over this value, biofilm accumulation increased with increasing roughness [34]. Surface roughness can, however, be measured in many ways. R_a_ gives an arithmetic mean of the surface roughness, while the topography and maximum peak to valley can differ significantly among materials with similar R_a_ values. Nanoscale morphology has also been shown to influence the adhesion of proteins and subsequent formation of biofilm in *in vitro* models [39]. An absolute threshold value measured in R_a_ in probably not clinically relevant for all materials, but gives a general indication of the clinical acceptance level. Furthermore, the surface roughness of most materials will increase over time in the oral cavity.

### 4.3. Chemical Composition

The chemical composition of the dental material will further affect the bacterial adhesion since both proteins and microorganisms can chemically attach or attract to components in the material, by van der Waal forces, acid-base reactions or electrostatic interactions [40]. In most patients, there will be several different materials present in the mouth simultaneously with can interfere with the biofilm formation and the microbiota in general. The chemical interaction between material and microorganisms can lead to alterations in the surface properties over time.

### 4.4. Dental Restorations

**Figure 5:**
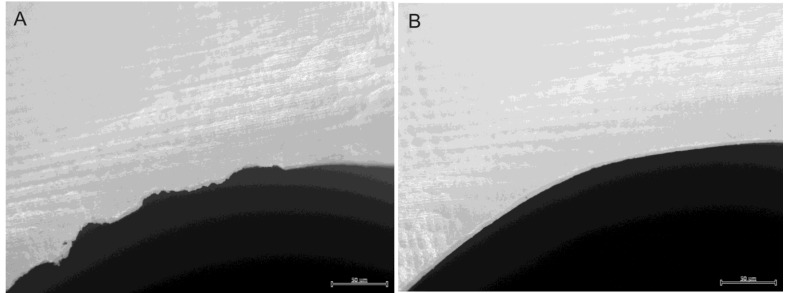
(**A**) A zirconia ceramic crown margin severely damaged by the machining procedure creating an uneven and biofilm-retaining margin between restoration and tooth; (**B**) A zirconia ceramic crown with a smooth undamaged crown margin that is easily cleaned. (photo: Marit Øilo).

The type of biomaterial in a dental restoration, can also affect the biofilm formation to a large extent [16]. Crowns and bridges are usually made of ceramics, metals or a combination of these. Ceramic materials have a smooth, polished surface which is easily cleaned. This is very good with regard to daily removal of the biofilm. Some ceramics have, however, very uneven crown margins from the machining procedures (Figure 5). A crown margin with many small defects will retain more plaque and bacteria than a smooth margin. Most metals can be polished to give very little retention for biofilms, although some alloys have a higher affinity to bacteria than others [41]. It seems that some bacteria are attracted to electrical charges in some alloys [16]. Biofilms on gold restorations, however, generally have low viability [41]. Some microbes are affected by the eluates from the metals. The mercury leaching in very small amounts from fresh amalgam restorations has, for instance, a bacteriostatic effect [41]. The border between the tooth and the restorations will always be problematic, since it is virtually impossible to make restorations with perfect adaptations all the way around [42,43]. The grooves or edges created by this will increase accumulation of plaque and complicate the mechanical removal of it afterwards.

**Figure 6:**
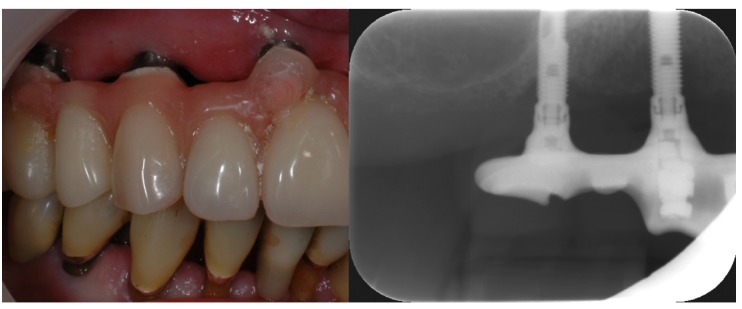
Implant in the upper jaw supporting a fixed denture. The rough area on the molar region implant is exposed due to previous periimplantitis (photo: Marit Øilo).

Dental implants are most commonly made of titanium which are inserted into the alveolar bone to function as artificial roots [35]. They penetrate the oral mucosa and are therefore exposed to the oral microflora. Implants usually have a smooth polished surface in the areas that are exposed to the oral cavity which can be easily cleaned. They are, however, often made of several small components that are fixed together with tiny screws [44]. There will always be gaps and crevices between these parts, creating almost a “greenhouse” for bacterial growth. Pathogenic bacteria in such gaps can cause inflammation in the surrounding bone or inhibit osseointegration [25,35]. Modern implants have a very rough surface in the part that is to be osseointegrated in the alveolar bone to enhance early bone attachment [35]. If the bone reacts inadequately or does not grow to cover the rough area, this will be exposed to the oral cavity as well. This surface will easily be colonized by oral bacteria which then will be almost impossible to remove completely (Figure 6). If pathogenic organisms are allowed to settle in such an area, it will be extremely difficult to arrest inflammation (periimplantitis). Since the implant is in direct contact with bone, the inflammation will potentially be able to spread via the bone or the blood faster than similar inflammation around teeth [16,35]. The teeth are separated from the alveolar bone by fibrous tissue, which work as a barrier to bacterial ingrowth. Currently, research cannot fully explain the differences between periimplantitis and periodontitis [45]. The microorganisms surrounding an implant with healthy supporting tissues are similar to those surrounding a healthy tooth [46]. However, periimplantitis locations seem to have more bacterial species and fungal species than locations associated with periodontitis. This is possibly an effect of the titanium in the implant.

**Figure 7:**
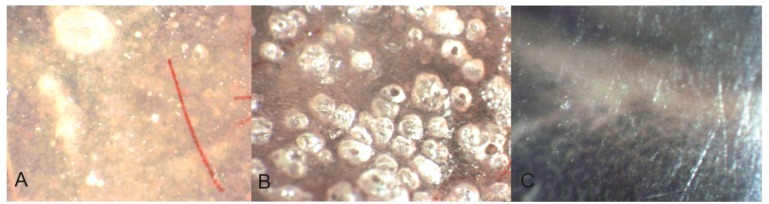
Different polymers have a different density and surface structure. The amount and size of pores and surface defects will affect the biofilm formation and composition. (**A**) polished heat cured acrylic; (**B**) unpolished cold cured acrylic; (**C**) polished polyamide (photo: Marit Øilo).

Polymers, such as resin based composites, glass ionomer and acrylics, have more pores and defects in the surface than metals, ceramics and enamel (Figure 7). Porosities will be filled with humidity and make perfect incubation chambers for certain microbes [14,24,47,48]. Biofilms on polymers develop quicker and will be more difficult to remove completely [41]. Acidic residues from the bacteria will roughen the surface, which will further complicate biofilm removal [15,16]. In order to achieve a surface roughness under the threshold value R_a_ = 0.2 µm on dental polymers special routines with stepwise polishing and finishing must be performed and repeated at regular intervals [38].

Removable dental prostheses, such as full or partial dentures or orthopedic appliances, will create new surfaces for biofilm formation, and thus increase the total amount of biofilm dramatically [30,49,50,51]. An increase of the total number of microorganisms alters the whole ecology of the oral cavity which can shift the balance between harmful and friendly microorganisms in the entire mouth [27]. The mucosa in direct contact with these appliances will be most affected, but the other structures in the mouth will be involved as well. Fungal species, such as *C. albicans*, can proliferate when the chemical and physical situations alter [24,52]. The oxygen level drops when large areas of the oral mucosa suddenly are covered by dentures. Organisms that demand oxygen (aerobic) will perish and make room for other species (anaerobic) [20,24,52,53,54]. Proliferations of *Candida* will cause an inflammatory response in the mucosa under the biofilm—denture stomatitis. The risk of re-colonization is great if the chemical and physical situation is not altered simultaneously with medical treatment [20,53,54,55]. The host’s health and nutritional status are important factors for both development and the possibility to cure this infection [1,6].

Dentures rest directly on the oral mucosa. The stiff and hard acrylic dentures create wounds or irritations in the oral mucosa if they do not have an optimal fit. These irritated sites are easily colonized by fungi and bacteria causing inflammation and pain. These problems are either treated with adjustments in the acrylic, which very often results in a rougher surface or by lining the denture with a material to either increase fit or softness [56]. The liner materials available are cold cured acrylic, polyvinyl siloxanes, or acrylics containing plasticizers. All these are much more porous than heat cured acrylics and have increased problems with fungal infections [47,57,58]. The softer materials are most problematic but also most comfortable for the patients. Patients with poor general health are most exposed, firstly, because they usually have more problems with wearing dentures, and secondly, because they are more susceptible for infectious diseases [59]. Some soft denture materials, such as polyamides are probably less prone to biofilm formation than acrylics due to fewer pores [60].

## 5. Future Aspects and Current Research

Due to the many problems associated with biofilm formation on dental biomaterials, the challenge is to improve the materials in use. Reduced polymerization shrinkage of dental resin composites is believed to reduce the leakage of microorganisms into the gap and thereby reduce the rate of secondary caries, although the clinical relevance is difficult to detect [61]. Fluoride release from glass ionomer cements are meant to reduce the demineralization of the dentine and enamel on the tooth adjacent to the cement [62]. Fluoride release from other polymers have not been equally successful. Lately, there have been many attempts at altering the chemical composition in the materials with the intention of reducing biofilm formation [4,63,64]. Different strategies are used to achieve this, mainly reducing the initial attachment of bacteria by altering the surface properties or reducing the viability of the attached bacteria by chemical components [4]. Some dental materials include monomers with antibacterial eluates [65]. Antibacterial chemicals, such as chlorhexidine or silver nanoparticles, can be embedded in nanoparticles in resin, coatings, acrylics, sealers or cements [66,67]. The nanoparticles dissolve slowly from the material and destroy the bacteria in the biofilm attached to the surface of the material. More than 50 abstracts each year concerning antibacterial effects of dental restorative materials have been presented in the International Association of Dental Research meetings worldwide in recent years, illustrating the current interest for this clinical problem. There are several potential benefits from including slow releasing antibacterial particles in biomaterials. The number of bacteria in the biofilm can be reduced, the potential harm from biofilm formation can be reduced, and the oral environment be made healthier. The antibacterial effect can be used in specific localizations, such as in the layer between filling and tooth, where they are most effective and thus reduce the general effect on the patient [68,69]. Antibacterial liners can ensure that remaining bacteria in the caries lesion are killed and thereby arrest the development of the lesion without excessive removal of tooth substances [68,69]. Antibacterial sealers for endodontic treatment can probably reduce the number of visits and increase success rates of endodontic treatment [70]. Antibacterial surface modifications or coatings are investigated for a wide range of applications also outside medicine and dentistry [4].

There are several uncertainties regarding the long term effects regarding surface coatings or slow releasing particles which have not been fully investigated. Coatings will be worn, roughened and may not have sufficient mechanical properties over time. Gradual release of particles will eventually alter the mechanical properties of the material. Such release can be compared with corrosion. Corrosion will normally result in increased surface roughness and loss of substance and thereby increase the retention for bacteria. It is likely that the release of nanoparticles over a long time will reduce the fracture strength, surface hardness, and wear resistance of the material. An antimicrobial layer between tooth and restoration may reduce both initial and long term retention by altering the chemical adhesion between layers. These potential side effects are rarely discussed or evaluated in the studies available. The effect of any antibacterial chemical will always be dose-dependent. It will be difficult to ensure that the dose of the released particles is high enough to be effective, while not weakening the material. It is reasonable to assume that the effect will diminish over time. Additionally, there may be a serious risk of developing multi-resistant bacteria [71,72] or other adverse reactions to the released particles [73].
